# A Comprehensive Review of Computer-Aided Models for Breast Cancer Diagnosis Using Histopathology Images

**DOI:** 10.3390/bioengineering10111289

**Published:** 2023-11-07

**Authors:** Alberto Labrada, Buket D. Barkana

**Affiliations:** 1 Department of Electrical Engineering, The University of Bridgeport, Bridgeport, CT 06604, USA; alabrada@my.bridgeport.edu; 2 Department of Biomedical Engineering, The University of Akron, Akron, OH 44325, USA

**Keywords:** review, breast cancer, computer-aided diagnosis (CAD), machine learning, deformable modes, classification, histopathology

## Abstract

Breast cancer is the second most common cancer in women who are mainly middle-aged and older. The American Cancer Society reported that the average risk of developing breast cancer sometime in their life is about 13%, and this incident rate has increased by 0.5% per year in recent years. A biopsy is done when screening tests and imaging results show suspicious breast changes. Advancements in computer-aided system capabilities and performance have fueled research using histopathology images in cancer diagnosis. Advances in machine learning and deep neural networks have tremendously increased the number of studies developing computerized detection and classification models. The dataset-dependent nature and trial-and-error approach of the deep networks’ performance produced varying results in the literature. This work comprehensively reviews the studies published between 2010 and 2022 regarding commonly used public-domain datasets and methodologies used in preprocessing, segmentation, feature engineering, machine-learning approaches, classifiers, and performance metrics.

## 1. Introduction

Breast cancer is projected to account for 1 in 3 new female cancers yearly in the United States (US) [1]. The survival rate for breast cancer is measured in 5-year intervals, considered relative survival rates, and does not consider the cause of death. The ACS study reported that the 5-year survival rate is 90%, the 10-year survival rate is 84%, and the 15-year survival rate is 80% [2]. According to the American Cancer Society (ACS) in the US, an estimated 287,850 new cases of invasive breast cancer will be diagnosed in women in 2022. In addition, 51,400 new cases of ductal carcinoma in situ (DCIS) are expected to be diagnosed, and approximately 43,250 deaths would occur in US women [1]. The World Health Organization (WHO) reported that breast cancer accounted for 12% of all new annual cancer cases worldwide and had become the most common form of cancer diagnosed globally as of 2021 [3]. The latest statistics state that an estimated 684,996 women died of breast cancer worldwide in 2020, and 2,261,419 new breast cancer cases were diagnosed worldwide in 2020 [4].

With technological advancements and healthcare systems, breast cancer survival rates have increased. Many variables can affect the survival rate of someone diagnosed with breast cancer. Most importantly, an early diagnosis can immensely increase the chances of survival. Recent technological advances have allowed for computer-aided detection methods to assist in diagnosing this form of cancer. The systems and tools commonly incorporated into cancer diagnosis are mammograms, ultrasounds, magnetic resonance imaging (MRI), and histopathology images.

Especially in the last five years, most CAD systems have been designed using supervised machine learning models via deep neural networks. While deep networks have many advantages, they have limitations and drawbacks. Database quality and size, high computational cost, overfitting, and black-box approach-related challenges must be improved and better understood. Breast cancer has become one of the most frequently studied fields. Comprehensive review papers evaluating recent works are valuable in presenting, comparing, and discussing the impacts of those works and forecasting future trends.

The existing review papers in the literature on breast cancer and histopathology focused on image analysis methodologies [5], epidemiology, risk factors, classification, detection, markers, and treatment strategies [6,7,8], a combination of different image modalities [9], cut-off levels of Ki-67 [10,11], and only deep neural network models [12,13,14]. The work in [15] surveyed the trends for breast cancer CAD systems but did not cover all stages of CAD systems. Our review exclusively focuses on all stages of CAD systems modeled for breast cancer using histopathology images. We reviewed each stage by reporting and evaluating the developed and/or employed techniques since 2012.

### 1.1. Scope of the Review

This review aims to compile data about the CAD systems using breast histopathology images, including datasets, preprocessing, segmentation, feature engineering, classification, and performance metrics. The primary purpose of the review is to seek answers to the following questions:

(a) Which histopathological image datasets are widely used in breast CAD systems?
(b) What are the preprocessing methods and their impact on the CAD systems?
(c) What are the employed segmentation and feature extraction methods?
(d) What are the most common performance metrics used?
(e) What are the trending methodologies and associated challenges in the field?

### 1.2. Article Selection Criteria

**Figure 1:**
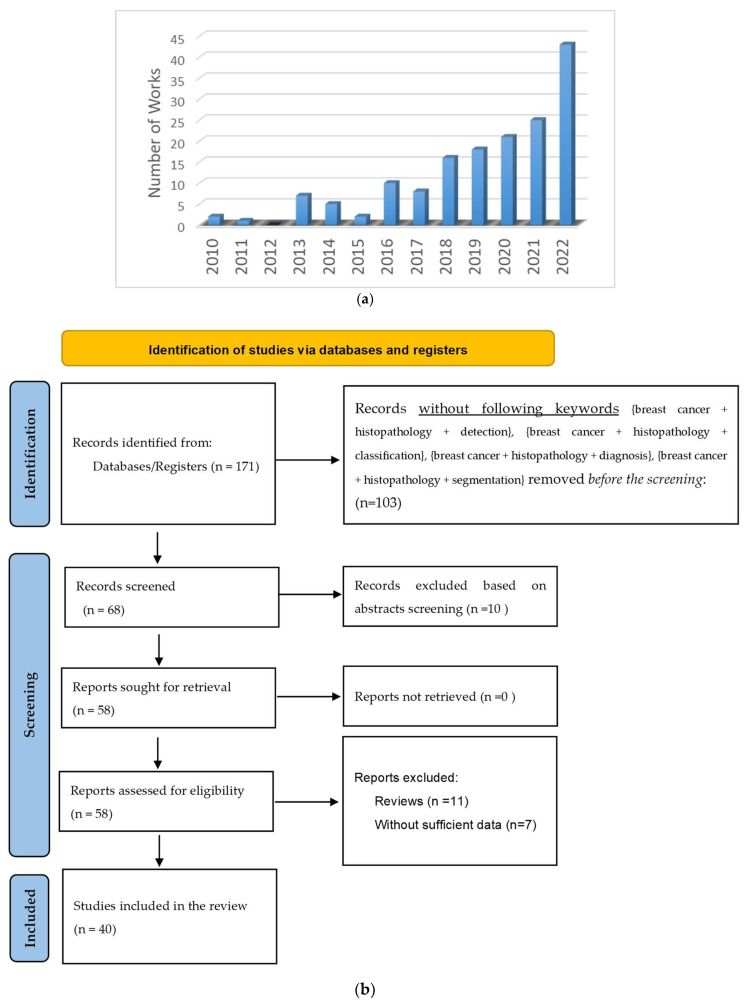
(**a**) The number of studies in CADe and CADx systems using breast histopathology images. (**b**) PRISMA flow diagram for the review of CADe and CADx systems using breast histopathology images.

We searched the most significant works in the literature between 2010 and 2022 by using the following keywords: {Histopathology}; {Breast cancer}; {Image analysis}; {Image processing}; {Histopathological image analysis}; {Computer-assisted Diagnosis}; {Digital pathology}; {Nuclei segmentation}; {Breast histopathology images}; {Automatic image classification}; {Breast biopsy}; {Histopathology image segmentation}; {Image classification}; {Carcinoma cancer}; {Breast cancer screening techniques}; {Medical image processing}; {Breast cancer detection}; {Computer-aided diagnosis (CAD)}; {Computer vision}; {Image recognition}; {Medical image classification}; {Pattern recognition and classification}; {Invasive ductal carcinoma prediction}; {Mitotic cell count}; {Digital pathology}; {Bioinformatics}; {Computational biology}. Peer-reviewed journal and conference papers were collected from credible search engines, including PubMed, IEEE, Elsevier, Wiley, Springer, etc. Only the studies with the following keywords: {breast cancer + histopathology + detection}, {breast cancer + histopathology + classification}, {breast cancer + histopathology + diagnosis}, {breast cancer + histopathology + segmentation} were considered in the review. Furthermore, we excluded the articles if they appeared in multiple sources, had a pure medical science focus, did not propose a CAD system, had no reported results, or were review papers. Figure 1a depicts the number of studies conducted between 2010 and 2022. There has been a significant increase in research work over the years. In 2022, the number of studies per year almost doubled. The PRISM (preferred reporting items for systematic reviews and meta-analyses) guidelines [16] for the review are presented in Figure 1b.

**Figure 2:**
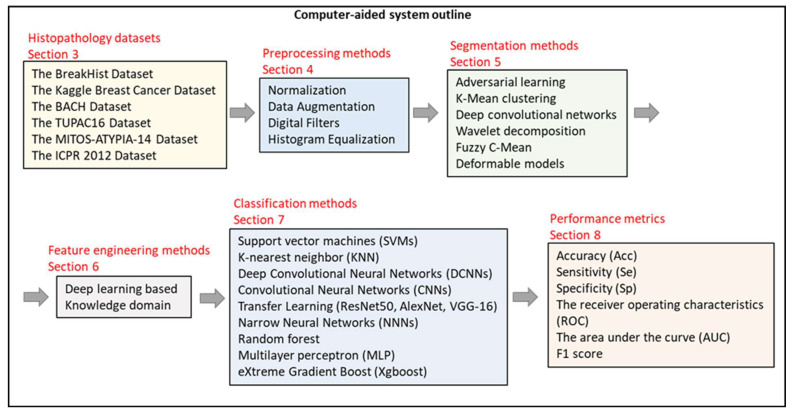
Organization of the review of analysis and diagnosis of breast cancer from histopathology images.

We summarized the most used breast histopathology image datasets in Section 3. The preprocessing methods employed in the reviewed works were explained in Section 4. Segmentation and feature engineering algorithms were presented in Section 5 and Section 6. Classification methods and performance metrics were covered in Section 7 and Section 8. Figure 2 presents the organization of our review process.

## 2. Basics and Background

Mammography techniques have been a diagnostic tool since the 1960s, and the ACS has officially recommended them since 1976 [17]. A mammogram uses low-dose amplitude X-rays to examine the breast [18]. The X-rays are part of a screening process and typically involve several breast X-rays. Mammograms show tumors and microcalcifications that may indicate cancer [18]. Mammography has aided in decreasing the mortality rate in women with breast cancer by 25–30% compared to a control group spanning 5 to 7 years [19]. It is reported that the doses of radiation required to produce mammograms are considerably low [18].

The use of ultrasounds in breast imaging dates back to 1951, when Wild and Neal described the characteristics of two breast tumors, one benign and one malignant, in an intact human breast [20]. In breast ultrasounds, sound waves are used, and their echoes can construct computer representations of the inside of a breast. A transducer device moves over the skin and sends sound waves that bounce off breast tissue. The transducer then picks up the reflected sound waves and uses them to construct 2D images. They can detect changes in the breast, such as fluid-filled cysts [21,22]. The efficacy of ultrasound screening alone in asymptomatic women will likely cause false positive and negative results. Therefore, a mammogram with an automated whole breast ultrasound (AWBU) is better in cases of dense-breasted women. According to a study by Kelly et al., 87% of cancer detections aided by AWBU were found in 68% of studies of women with dense breasts [19,23]. Ultrasounds can also be used in breast cancer detection, specifically when guiding a biopsy needle into a region of interest in the breast for cells to be taken out and tested for cancer. Unlike mammograms, an ultrasound introduces minimal risk to a patient because it does not expose a person to radiation [22].

The breast MRI was first brought into use in the late 1980s. According to a study in 1986 by Heywang et al., preliminary results indicated that an MRI of breasts using gadolinium administration showed increased enhancement relative to normal breast tissue [24]. In an MRI, the hydrogen nucleus, abundant in water and fat, is used for imaging. The magnetic property of the nucleus is used in conjunction with radio waves and strong magnets, creating a detailed picture of the inside of a breast [19,25]. Breast MRI is typically used for women at high risk for breast cancer. It is usually paired with a mammogram because an MRI alone can miss specific cancers that can be found with a mammogram. Once cancer has been diagnosed, a breast MRI can be done to help localize the cancer, determine its exact size, and look for other tumors in the breast. Unlike mammograms, an MRI uses strong magnets instead of radiation to make detailed cross-sectional pictures of the body by taking pictures from different angles. Therefore, there is no exposure to radiation during this procedure [25].

**Figure 3:**
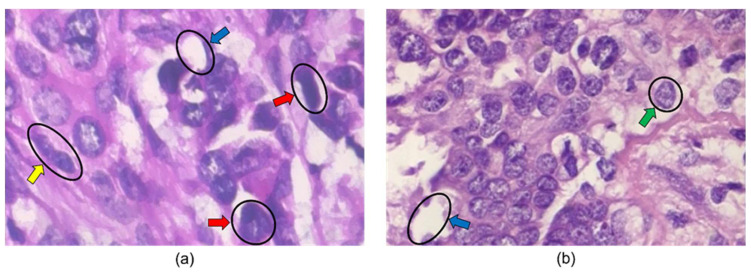
Histopathology images from BreakHis 400× dataset. The blue, green, yellow, and red arrows indicate adipose tissue, a cell nucleus, a mitotic figure, and large nuclei in the images. The image in (**a**) is labeled malignant, while the image in (**b**) is labeled benign.

The first breast biopsies were performed in the 1850s and 1860s by Skey, Sir James Paget, and John Eric Erichsen [26]. A biopsy involves a physician removing small pieces of breast tissue from areas of interest that can be further analyzed in a laboratory to determine if cancer cells are present [27]. A breast biopsy is usually ordered to check a palpable lump or mass, examine a problem seen on a mammogram, and determine whether a breast lump or mass is either malignant or benign [28]. The diagnoses are carried out by pathologists looking at histopathology images and examining them for signs indicating benign or malignant cancer. Biopsy extraction techniques are ultrasound-guided, mammographic-stereotactic-guided, magnetic resonance-guided, fine-needle aspiration, core needle, vacuum-assisted core, and surgical biopsy [28,29]. See Figure 3.

Examining many histopathological images is cumbersome and time-intensive for pathologists, and it can result in a certain margin of human error. Due to these reasons, computer-aided detection (CADe) and computer-aided diagnosis (CADx) systems help assist physicians and experts in increasing the success rate of the analysis/diagnosis. The role of a CADe system focuses on the localization of a specific object or region of interest (ROI), as the particular area of interest is specific to the task. In the case of breast cancer research, detection will be geared specifically towards the nuclei present in a histopathology image, which will then be segmented to make up the ROIs in the images. The CADx systems can extract and analyze features in segmented images and use classifiers to measure and distinguish between benignity and malignancy [30].

## 3. Histopathology Image Datasets

The breast cancer histopathological image classification (BreakHis), the Kaggle breast cancer histopathology images dataset, the ICIAR 2018 grand challenge on breast cancer histology images (BACH) dataset, the tumor proliferation assessment challenge 2016 (TUPAC16), the MITOS-ATYPIA-14 challenge, and the international conference on pattern recognition (ICPR 2012) dataset are the most widely used datasets in the literature. Table 1 lists the datasets and their URLs.

### 3.1. The BreakHis Dataset

The BreakHis dataset was built with the P&D Laboratory for Pathological Anatomy and Cytopathology in Parana, Brazil. Tissue samples that comprise this dataset were generated from breast biopsy slides collected by the surgical (open) biopsy method. Then the extracted samples were stained with hematoxylin and eosin (H&E) [31]. The 9109 microscopic images, 5240 malignant and 2480 benign, were sampled from 82 patients of different magnifications (40×, 100×, 200×, and 400×) [32]. “The dataset currently contains four distinct histological types of benign breast tumors: adenosis (A), fibroadenoma (F), phyllodes tumor (PT), and tubular adenoma (TA); and four malignant tumors (breast cancer): carcinoma (DC), lobular carcinoma (LC), mucinous carcinoma (MC), and papillary carcinoma (PC) [32]”.

Most of the CAD systems are modeled using the 400× dataset. Table 2 portrays the dataset distribution by magnification for the BreakHis dataset.

### 3.2. The Kaggle Breast Cancer

The Kaggle histopathology images dataset is a commonly sourced dataset for breast cancer research consisting of benign and malignant IDC cases. The dataset comprises 162 whole-mount slide images of breast cancer samples with a magnification of 40×. A total of 277,524 patches were sectioned out from the entire mount slide images, each with a size of 50 × 50. A total of 198,738 images in the dataset test negative for IDC, and 78,786 images test positive (Table 3). The images used for this dataset were each associated with a patient ID and label marked by pathologists that indicated whether the patient was positive or negative for IDC [33].

### 3.3. The ICIAR 2018 Grand Challenge on Breast Cancer Histology Images (BACH) Dataset

The BACH dataset is widely used in breast cancer research and was organized to promote methods for automatically classifying breast cancer biopsies [34]. A collection of 400 labeled H&E-stained breast histology microscopy images and ten pixel-wise labeled and 30 non-labeled whole-slide images make up this database. Expert pathologists annotated the microscopy images from the Institute of Molecular Pathology and Immunology of the University of Porto and the Institute for Research and Innovation in Health. Whole-slide images were annotated by a pathologist and revised by a second expert [34]. In Table 4, microscopy images are classified as follows: 100 normal, 100 benign, 100 in situ carcinomas, and 100 invasive carcinomas [35].

### 3.4. The TUPAC16 Dataset

The TUPAC16 set consists of 821 whole-slide images from the Cancer Genome Atlas (TCGA) network (Table 5). The images are randomly separated into 500 for training and 321 for testing. Two types of tumor proliferation data are available for the images, including a mitotic score involving a manual count of mitosis occurrences performed by a pathologist and a PAM50 proliferation score based on molecular data [36,37].

### 3.5. The MITOS-ATYPIA-14 Dataset

The MITOS-ATYPIS-14 set was constructed for mitosis detection (mitotic count) and the evaluation of nuclear atypia (nuclear pleomorphism), which are essential parameters for diagnosing breast cancer [38]. The set of biopsy slides for this dataset is stained with H&E and was annotated by Frédérique Capron, head of the Pathology Department at Pitié-Salpêtrière Hospital in Paris, France. Several regions at 20× magnification were selected and used for scoring nuclear atypia (atypia scores of 1, 2, and 3) within the slides. Scores 1, 2, and 3 denote low, moderate, and high-grade atypia. Then, the 20× regions were divided into four frames at 40× magnification and used to annotate the mitotic figures to arrive at a mitotic count for the image. The dataset consists of 284 frames at 20× magnification and 1136 frames at 40× magnification (Table 6) [38]. The dimensions of the frames are also provided in the dataset: Aperio Scanscope XT and Hamamatsu Nanozoomer 2.0 HT.

### 3.6. The ICPR 2012 Dataset

The ICPR 2012 dataset was provided by Professor Frédérique Capron’s team in the pathology department at Pitié-Salpêtrière Hospital in Paris, France. Five slides of breast cancer were stained with H&E and scanned using three different pieces of equipment: the Aperio ScanScope XT slide scanner (ASXT), the Hamamatsu NanoZoomer 2.0-HT slide scanner (HNZ), and the ten-band multispectral microscope (MSM) [39]. The miotic figures in the image were annotated manually by a pathologist. Using five different slides scanned at 40× magnification, ten high power fields (HPF) per slide make up 50 HPFs comprising the dataset. The total number of mitotic cells in the 50 HPF for both scanners is 326 and 322 mitotic cells using the multispectral microscope (Table 7) [39].

## 4. Preprocessing Methods

The preprocessing stage for any work is considered one of the essential stages for a body of work after image acquisition. Raw images may not adequately portray the specific features of interest to the research. Therefore, one of the goals of the preprocessing stage is to make the region of interest more suitable for analysis. Normalization, data augmentation, digital filters, and histogram equalization are commonly used preprocessing techniques.

### 4.1. Normalization

**Figure 4:**
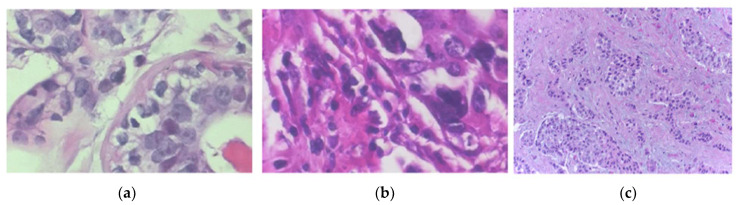
Images in (**a**,**b**) are from the BreakHis ×400 dataset. The image in (**c**) is from the BACH dataset. Images show various stain colors and illuminations.

Normalization techniques play a significant role in preprocessing as they adjust image attributes. The normalization techniques include stain color normalization, global contrast normalization, and illuminant normalization [40,41,42,43,44,45,46,47,48]. When dealing with H&E-stained images, in particular, the variability in the appearance of the images can affect the algorithms’ performance. These irregularities can come from the tissue preparation and staining processes used by different labs, including but not limited to the antigen concentration, incubation time and temperature, and slide digitization conditions, including differences in optics, light detectors, or light detectors used in the scanners [5,49]. Kashyap et al. utilized stain normalization to deal with the volatile expression of H&E images that exhibited the same malignancy level. The process improved the contrast and brightness of the images using a contrast-limited adaptive histogram equalization method without compromising any of the information from the image [40]. Figure 4 shows images with various stain colors and illuminations.

Noumah et al. adapted the Vahadane method as a preprocessing stage to solve the stain variability issue with the BreakHis dataset. The technique was advantageous because it allowed for the transformation of one image into another while preserving the color values of the original image.

Furthermore, it preserved biological structure information by modeling stain density maps based on non-negativity, sparsity, and soft classification [41]. Vo et al. used a logarithmic transformation technique to compute the image’s optical density, followed by the singular value decomposition method (SVD) on the optical image density image to estimate relevant degrees of freedom and construct a 2D projection matrix. This method transformed images into a common space and reduced inconsistencies [45]. Kausar et al. proposed a stain color normalization technique that condensed the stain variations using stain vectors and concentration maps. Stain normalization and color deconvolution were applied to the target, training, and testing images. Using the averages of the target stain vector and concentration map, they constructed a normalization function, allowing the color distribution of the training and testing images to be mapped onto the target image [46].

### 4.2. Data Augmentation

Data augmentation is used to increase the size of image datasets as machine learning (ML) algorithms require large datasets for training [40,41,42,43,44,45,46,48,50,51,52]. Some of the more commonly used methods for data augmentation involve image transformations and color modifications. Image transformations can include rotations, reflections, scaling, and shearing. Color modifications include but are not limited to histogram equalization, enhancing contrast or brightness, white balancing, sharpening, and blurring [53].

A recent study used data augmentation to increase the number of images. To overcome the overfitting, they integrated the BreakHis and BreCaHAD datasets and performed data augmentation to obtain a more robust dataset using flipping, rotating, shifting, resizing, and gamma correction. The scaling factors used on the images were 0.5×, 0.8×, and 1.2× for each image. Horizontal and vertical transformations generated images with 40-, 80-, 120-, and 180-degree rotations. After applying 19 parameters to 7909 sample images, the number of images was increased to 153,349 total images. The study reported the evaluation metrics for the original and augmented datasets. The accuracy achieved using the augmented datasets was reported to be about 5% and 3% higher than the original BreakHis and BreCaHAD datasets, respectively [40].

Noumah et al. implemented data augmentation methods to expand the training data set size by using random zoom augmentation with a value 2, random rotation augmentation with a value of 90°, and horizontal and vertical flip augmentation [4]. Boumaraf et al. classified histopathological breast cancer images via a magnification-dependent and independent-based approach in 2021. Using a three-fold data augmentation method, the training set was artificially tripled in size by employing three random transformations: a random horizontal flip, a random vertical flip, and a random rotation with 40 degrees [42].

Kate and Shukla introduced a novel method for the automatic classification of histopathological images of breast cancer using the deep learning model ImageNet. The scarcity of data was overcome by implementing different geometric transformations to train this deep learning network properly. The size of the training set was tripled by using random transformations such as random vertical flips, random horizontal flips, and random rotations [43].

Hameed et al. classified breast cancer histopathology images using an ensemble of deep-learning models. Batches of tensor image data were generated using the ImageDataGenerator Keras deep learning library provided while implementing real-time data augmentation. Images that were administered to the generator were transformed by a manner of random translations and rotations. The random rotation is specified by a rotation range between [−40 and 40] degrees. Also implemented into these transformations was a width and height shift, where the image was shifted either up or down or between left and right. If, for a reason, a rotation of the image caused pixels from the original image to become out of frame, a ‘reflect mode’ was used to fill the empty pixels [44].

Vo et al. increased the amount of training data by implementing data augmentation techniques. Performing geometric augmentations, which included reflecting, randomly cropping, rotating, and translating the images, were among the changes made to the existing images [45]. Kausar et al. implemented data augmentation techniques to increase 500 H&E-stained images to 16,575 images. Morphology and color invariances were achieved by rotating, scaling, elastic deformation, and channel color modification techniques [46]. Rakhlin et al. performed 50 random color augmentations on each image and downscaled the images in half to 1024 × 768 pixels from the original size. The downscaled images were cropped down to 400 × 400 and 650 × 650 pixels [48]. Romano et al. augmented images by using a random rotation range of 0 to 20 degrees along with width and height shifts ranging from a fraction of 0.20 to the total width or height of the image. The alterations also consisted of random horizontal and vertical flips [50]. Chang et al. applied augmentation techniques, including rotating the images by 90, 180, and 270 degrees and mirroring and randomly distorting images. The original dataset was augmented to 11,184 images from 1398 images in the original dataset [51].

Yari et al. applied deep learning techniques to arrive at a diagnosis for breast cancer. By implementing data augmentation techniques, they were able to boost the CAD system’s performance. This was arrived at by first resizing the images to 224 × 224 pixels, randomly flipping some horizontally, and randomly rotating and cropping some images. Color jitter for images is also used to change the tone of the original color based on hue, saturation, and value [52].

### 4.3. Digital Filters

Digital filters are designed to reduce or remove noise and artifacts in an image [54,55,56]. Hirra et al. classified histopathological images using patch-based deep-learning modeling. Using a Gaussian filter with a fixed kernel size, they could control the smoothness of the images and reduce the weight of blurring pixels [54]. Vaka et al. detected cancer by leveraging machine learning. As part of the preprocessing phase, a Gaussian filter was used for noise removal [55]. A study out of Jalpaiguri Government Engineering College in India used a deep residual neural network to detect breast cancer in histopathology images. The Gaussian blur algorithm was used for the denoising of images with low resolutions to reduce regions specifically affected by the noise [56].

### 4.4. Histogram Equalization

Histogram equalization and logarithmic transformation are also widely used preprocessing techniques [45,46,57,58]. Narayanan et al. used a convolutional neural network to classify histopathology images. Histogram equalization was used to adjust the image’s contrast by using its histogram and served as a method to study the algorithm’s performance. In addition to histogram equalization, a color constancy method was applied to the image to input the convolutional layers [57]. Vo et al. used logarithmic transformation to compute the optical density for each histology image to implement the method of stain normalization [45]. Kausar et al. used a wavelet transform, which was used to decompose the images into a set of frames that relayed important information about the spatial and frequency characteristics of the images [46]. Jiang et al. applied histogram equalization to the images after performing a color space transform on the images. A logarithmic transformation was also used to convert the image’s color to an optical density [58].

## 5. Segmentation Methods

Image segmentation is a technique for dividing a digital image into segments, which can simplify further processing or analysis of the image. It involves assigning labels to pixels to identify objects, people, or other stages. It is commonly used in object detection, where an algorithm finds objects of interest in an image. The object detector then operates on a bounding box defined by the segmentation algorithm, improving accuracy and reducing inference time. Image segmentation is a key building block of computer vision technologies and algorithms. It is used for many practical applications, including medical image analysis, autonomous vehicles, face recognition, video surveillance, and satellite image analysis [59]. In breast cancer research, segmentation plays an important role, especially when segmenting the nuclei, because extracted features can indicate whether the cells in the histopathology image are undergoing mitosis. However, segmentation of histopathological images is a challenging task because of the varying characteristics of the images, including the magnification factor, resolution, and image quality.

Methods applied in this field for segmentation include but are not limited to adversarial learning, K-Mean clustering, deep convolutional networks, wavelet decomposition, and Fuzzy C-Mean [34,55,60,61,62,63,64,65,66].

Lin et al. used adversarial learning with data selection for segmenting breast cancer in histopathological images. One segmentor and two discriminators comprise the adversarial learning framework. The segmentor generates segmentation outputs for the source and target domains, while the discriminator distinguishes whether the outputs are from the source domain or the target domain. The Deeplab_V2 structure was used as a segmentation network, with ResNet101 as the basis. The atrous spatial pyramid pooling (ASPP) module was used to encode multiscale information in feature maps in conjunction with an un-sampling layer with softmax output responsible for up-sampling the output to the input dimensions. The segmentation network is optimized using the segmentor and discriminators trained simultaneously [60].

Li et al. focused their research on the classification of breast histopathology images with a ductal instance-oriented pipeline, which consisted of a duct-level instance segmentation model, a tissue-level semantic segmentation model, and three levels of features for diagnostic classification. The process for this segmentation begins by feeding the input ROI to the duct-level and tissue-level segmentation modules to produce instances of both the duct-level and tissue-level segmentation masks. For the instance segmentation network, after the ROI has produced the duct candidates, they are classified based on whether or not they are ducts, and a bounding box is also constructed along with a pixel-wise mask of the duct. An off-the-shelf segmentation network was applied for semantic segmentation that splits the input image into non-overlapping regions. It can predict a segmentation mask for the different regions using a resolution encoder-decoder structure [61].

Tan et al. proposed an automated framework that quantifies tumor regions using a spatial neighborhood intensity constraint (SNIC) clustering approach and fuzzy C-mean (FCM). As part of the clustering stage, centroids of the FCM are generated based on domain knowledge using knowledge-based initial centroids selection. This process reduces the search space and limitations of conventional FCM, such as dead center and center redundancy. The function of the SNIC is to eliminate the nucleus cells from the image while preserving the information and eliminating the fuzziness of the image. The K-mean clustering algorithm uses the cyan channel to segment the nucleus cell. Then, it is used as a mask to remove the pixels of the nucleus cells in the RGB images by setting the RGB intensity values of each pixel corresponding to the nucleus to 0 [62].

Sebai et al. employed partially supervised semantic segmentation for mitosis detection by using two-stream fully convolutional networks consisting of a large, weakly annotated mitosis dataset and a small, fully labeled mitosis dataset. The score maps of the two FCNs were fused to obtain more accurate mitosis detection. The fusion was followed by integrating an easy-to-train weight transfer function that allowed for the transfer of semantic knowledge from the segmentation branch trained with weak labels to another semantic segmentation branch trained with strong labels [63].

Priego-Torres et al. used a deep convolutional network to segment H&E-stained histopathology images automatically. Their method involved processing the whole-slide images into various patches and applying a deep convolutional neural network with an encoder-decoder with a separable atrous convolution architecture to the image patches. A fully connected conditional random field is then used to combine the local segmentation tiles while avoiding discontinuities and inconsistencies [64]. Vaka et al. proposed leveraging machine learning to aid in breast cancer detection. Their methodology produced better-quality images using a new deep neural network with a support value method. After removing the noise and extracting features from the preprocessed images, the breast tumors are segmented using histo-sigmoid-based fuzzy clustering [55].

Belsare et al. implemented a spatial-color-texture-based graph partition method to segment histopathology images. The spatial-color-based superpixel image representation is generated using a distance-based similarity function; then, the histology image and breast duct are partitioned using a texture classifier. Finally, the final segmented image is obtained using a graph portioning method in computer vision [65]. Wang et al. built a system to segment and classify nuclei in breast cancer histopathology images automatically. The CADx system initially performed a bottom-hat transform on the grayscale image to enhance the contrast between the cell nuclei and the background. The image’s ROIs are obtained using wavelet decomposition and multiscale region growth. Applying adaptive mathematical morphology and curvature scale space as part of a double strategy splitting model allows overlapping cells to be split for better accuracy and robustness [66].

Histograms and thresholding methods are other popular segmentation methods used in histopathology images. Kaushal and Singla [67] computed the energy curve to obtain tending thresholds and later evaluated the entropies for each tending threshold to find the best thresholding. The segmented regions were processed through morphological operations as a post-process. They reported the advantages of their work as incorporating spatial information, no prior setting of any initial parameter, magnification independence, and automatic determination of the inputs for morphological operations. A recent study developed a segmentation method to extract the morphological characteristics of lymphocytes [68] precisely. It differs from other studies by using the same network, called the dense dual-task network (DDTNet), for detecting and segmenting the lymphocyte. It reported compatible performance compared to state-of-the-art methodologies. For instance, DDTNet outperformed some known networks, including U-Net and HoVer-Net. The study reported a limitation since the detection and segmentation methods were bound to have the same errors as the traditional models. The robustness of the model is not yet generalized since the work was evaluated on small datasets.

Wahab et al. [69] employed an off-the-shelf, pre-trained deep CNN for the segmentation of mitosis. They used skip connections and demonstrated their effectiveness in fully convolutional networks as mitoses. Transfer learning-based mitosis segmentation (TL-Mit-Seg) was applied to the preprocessed images. Stain normalization, annotation, and cropping were applied to the raw images. To overcome the class imbalance, transfer learning produced a ratio of 1:12 on the validation set. The work did not use undersampling to solve the dataset imbalance problem since it might cause data loss. Skip connections were used in the residual learning, serving two purposes: reducing the effects of vanishing gradients and improving the spatial resolution of the segmented image.

## 6. Feature Engineering Methods

The feature engineering process is an integral part of CADx systems’ design. In breast cancer histopathology research, the engineered features are done with careful consideration of what distinguishes a cancer cell from a normal cell.

Rehman et al. used three different feature vector sets to distinguish between classes. The first feature vector set has 87 features and was useful in discriminating malignant cases based on the pattern difference. Information can be collected about the overall pattern by focusing on the texture of the whole patch. The second feature vector set carries a total of 28 features. This feature set was focused on determining the development stage of the nucleus, specifically by examining the circular shape around the nucleus and noting its irregular or regular circular shape. The third feature vector set had three features and focused on statistical features that could represent the pattern variation in each patch. Different cells in the image belonging to another class will exhibit variations in the pattern histograms. The mean value, peak, and variance can be extracted from the different histograms [70].

Kashyap et al. proposed a multiscale stochastic dilated convolution model capable of enhancing small and low-level features like edge, contour, and color. They could also remove redundant and similar features in the model that made the process more complex by using a series of linear operations on each intrinsic feature to generate ghost features [40].

The authors in [71] used parallel ‘same’ and ‘valid’ convolutional blocks (PSV-CB) to combine two forms of feature coding. An operational flow is made up of several ‘same’ convolutions and followed by strident max-pooling, which is known as hard feature coding. The operational flow uses step-by-step valid convolutions that reflect feature extraction and downsampling concurrently, known as ‘soft’ feature coding. Using the feature maps obtained from these operational flows, they could highlight pertinent content in the images.

Karthiga et al. applied a deep convolutional neural network for feature extraction in the initial stages of their methodology. Balancing the training data and the training iteration contributed to the overall accuracy of the classification rate. By supplying the deep learning model with a large dataset, they circumvent the alternative of using conventional machine learning techniques in conjunction with handcrafted features, resulting in less classification accuracy [72].

Li et al. used a ductal instance-oriented pipeline to classify breast histopathology images using three levels of pixel-wise features. Their work used a combination of histogram features, co-occurrence features, and structural features to extract features from tissue-level segmentation masks. The histogram features express the distribution of tissues in the image, co-occurrence features can encode spatial relationships, and structure features can extract frequencies from layers inside and outside of the duct instance and capture changes in the structure’s shape [61].

Hirra et al. classified histopathological images using a patch-based deep learning model. Features extracted for this study were done through an unsupervised method using feature vectors made up of features from the histopathology image patches. The features are learned automatically by creating image patches of the same size. The supervised portion of their method involves a learning phase that interprets the extracted feature matrix using a backpropagation neural network [54].

Labrada and Barkana [73] developed a feature set that would adequately represent the characteristics of the nuclei present in histopathology images by extracting geometrical, directional, and intensity-based features. Thirty-three features were extracted from each segmented region within the images. The geometrical set consisted of 5 features: area, perimeter, roundness, area-to-perimeter ratio, the ratio of the segmented region to the area of the fitting rectangle (AR_ratio), and the number of cells segmented in the image. The directional set used spatial distances in the segmented regions, measuring from the ROI’s center to the cell’s outstanding borders. A pixel count was performed to trace the eight cardinal directions (north, south, east, west, northeast, northwest, southeast, and southwest) to map the cell’s shape for analytical purposes. The center of the ROI was determined by enclosing the region in a bounded box and then taking the intersection point between both midpoints of the length and width. Their algorithm then calculated a mean, standard deviation, and range for each given direction while considering all of the ROIs in the particular image, totaling 24 features. The AR_ratio feature from the geometrical set addresses a specific concern from the directional set. It accounts for certain areas of an ROI that may not get appropriately mapped according to the directional mapping. The intensity-based feature set was composed of 3 features and focused on extracting information about the brightness of the ROI by looking at the pixel values of the ROIs and then calculating the mean, standard deviation, and range for a total of 3 features.

Wang et al. designed a classification system for breast cancer histopathology images based on deep feature fusion and enhanced routing. Their network consisted of two parallel channels that could extract convolution and capsule features simultaneously. The features were fused through a fusion method to combine into more discriminative features. Semantic features extracted by CNN and spatial features extracted by CapsNet are fused [74].

Kate and Shukla used the neural network ResNet-18 pre-trained on ImageNet to perform intrinsic feature learning. [43]. Vaka et al. implemented phylogenetic diversity in their work, often used to identify the distribution of a group of species and the relationship between species. Using this, the five features they defined were the sum of the phylogenetic branch lengths of each species, the sum of phylogenetic distances, the mean nearest neighbor distance, phylogenetic species variability, and phylogenetic richness [55].

Vo et al. classified breast cancer histopathology images using discriminative features trained by an ensemble of DCNNs. By implementing an ensemble, they increased the prediction accuracy rate. Multiscale input images were applied to the ensemble network and passed through at least one CNN. The ensemble network expands the receptive field of the original image, covers global features, and can extract multiscale local features [45].

Kausar et al. extracted in-depth features from Haar wavelet-decomposed images and used multiscale discriminative features to classify multiclass breast histopathology images. Using a feature concatenation strategy, they used a deep CNN model incorporating multiscale convolution features [46]. Rakhlin et al. used a LightGBM, a highly efficient gradient-boosting decision tree, for supervised classification. A 2-class and 4-class classification was performed for normal and benign non-cancerous cases versus in situ and invasive cancerous cases [48]. Wang et al. extracted shape features, including area, perimeter, eccentricity, roundness, and circularity. Statistical values, including mean, standard deviation, relative smoothness, skewness of the histogram, uniformity, and entropy, were obtained as textural features to analyze the spatial distribution of gray values [66].

## 7. Classification/Detection/Diagnosis Algorithms

**Figure 5:**
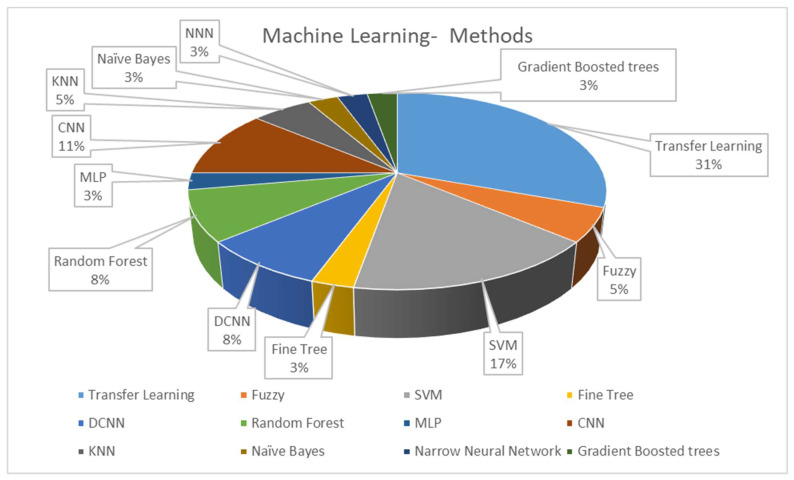
Distribution of the machine learning methods in CADe and CADx systems using breast histopathology images.

The classifier is a determining step in CADe and CADx systems’ algorithmic processes. After utilizing all the information acquired in the feature engineering process, classification algorithms can be trained for diagnosis and detection. Figure 5 illustrates the classifier approaches between 2010 and 2022.

The work in [70] implemented support vector machines, random forests, and Naïve Bayes classifiers as part of their classification architecture. Majority voting was applied to all three classification outputs after getting individual classifications from the classifiers to get the most accurate output. Their system performed well and adequately identified mitosis’s occurrence throughout the four different stages of mitosis. It had an accuracy of 86.38% for detecting mitotic cells in the MITO-ATYPIA 14 dataset.

Noumah et al. used colored stained images to develop an architecture that consisted of three pre-trained deep convolutional neural networks (DCNN) that worked in parallel. The output of each branch was passed onto a global average pooling layer, and the output of the three layers was then concatenated into one layer with 4640 neurons. Finally, dense layers were used to convert the 4640 neurons into two classes, either benign or malignant. Overall, their suggested model performed at an accuracy of 98% in determining the nature of a tumor [41].

Lin et al. proposed a framework comprising three stages: adversarial learning for domain adaptation, target domain data selection based on entropy, and model refinement with selected data and pseudo-labels. The atrous spatial pyramid pooling (ASPP) module was used to encode multiscale information into feature maps; this is directly followed by an upsampling layer with softmax output, which then upsamples the output dimensions to the input dimensions [60].

Jiang et al. used a specific classification task. They implemented it using an input-collaborative PSV-ConvNet that performs an end-to-end with no image color normalization and domain knowledge [71]. Yari et al. focused on a binary and multi-classification approach that could discern malignant and benign cases and different breast cancer types in the images. Their proposed model worked on magnification-dependent and magnification-independent classification methods and used ResNet50 transfer learning to supplement the low volume of the BreakHis dataset, which was not large enough for proper training. Using ResNet50 decreased the training error when implementing a standard optimization algorithm to train the network [75].

Karthiga et al. used the fine-tuned pre-trained models Alexnet and VGG-16 to achieve better performance classification. DCNN and transfer learning methods were also implemented for binary and multiclass classification. For the CNN, an architecture of 15 deep layers was used with learning parameters to implement the design [72]. Li et al. used various classifiers for their classification architecture, including a random forest model, a 3-degree polynomial SVM, an SVM with a radial basis function kernel, and a multilayer perception with four hidden layers. In binary classification, if the number of features was greater than the number of ROIs in a given task, principal component analysis (PCA) was performed to reduce the number of features to 20 dimensions. When classifying multiclass scenarios, a U-net extension with a separate branch for diagnostic classification was used [61].

Hirra et al. used fine-tuning as the second stage of deep belief network learning. During this portion of the learning, the model is assigned class labels. Then, they developed a model formed by the feature matrix of images from their design’s training portion to classify cancerous and non-cancerous regions. Logistic regression was used to classify the patches identified in the histopathology images [54].

Enhanced routing was used in [74] to assist in classification by optimizing routing coefficients indirectly and adaptively by modifying the loss function and embedding the routing process into the training process to learn routing coefficients.

Vaka et al. used SVM, random forest, multilayer perceptron (MLP), a type of deep artificial neural network, and eXtreme Gradient Boost (Xgboost), which is a library based on the gradient increase framework and can be used for regression and sorting [55].

Labrada and Barkana used four machine-learning algorithms to classify histopathology images from the BreakHis dataset, including decision trees, SVM, K-nearest neighbors, and narrow neural networks, in conjunction with PCA, to reduce the dimensionality of the dataset. The different feature sets were tested with each classifier, and their performance was recorded. Also, the feature sets were tested with each classifier as an entire group to gauge the performance of all feature sets working together. The most favorable result was obtained using all 33 features of the combined feature sets and a narrow neural network (NNN) that achieved an accuracy of 96.9% [73].

Yang et al. used a guided soft attention network to classify breast cancer histopathology images. A multi-task learning framework was implemented to design a CNN that could learn the ROI mask from the global image and guide the focus of the classification network [76].

Vo et al. used multiscale breast cell-extracted features and used them to train gradient-boosting tree classifiers. Combining the boosting tree classifiers with a DCNN achieved favorable classification results. A model combining majority voting and gradient-boosting trees achieved higher accuracy and sensitivity scores [45].

## 8. Performance Evaluation Metrics

Performance metrics are used to assess and validate the developed CAD systems. The histopathology datasets provide ground-truth labels for the benign or malignant tissues in the images. It makes it possible to calculate true positive (*TP*), true negative (*TN*), false positive (*FP*), and false negative (*FN*) metrics, which can be used to determine commonly used accuracy (Acc), sensitivity (*Se*), specificity (*Sp*), the receiver operating characteristics (ROC), the area under the curve (AUC), and the *F1* score. Although these metrics are well known, we find it proper to present the calculation formula for each of them here.

*TP* represents the image correctly classified as malignant,
*TN* represents the image correctly classified as benign,
*FP* represents the image falsely classified as malignant, and
*FN* represents the image falsely classified as benign.

(1) $$Acc=\frac{TP+TN}{TP+TN+FP+FN}$$

(2) $$Se=Recall=\frac{TP}{TP+FN}$$

(3) $$Sp=\frac{TN}{TN+FP}$$

(4) $$Precision=\frac{TP}{TP+FP}$$

(5) $$F1\;score=2\times \frac{Precision\times Recall}{Precision+Recall}$$

Improving the accuracy of the systems is a challenge without negatively impacting the precision and sensitivity of the systems. The higher sensitivity value means a higher value of the *TP* and a lower value of the *FN*. The lower sensitivity value means a lower value of the *TP* and a higher value of the *FN*. Sensitivity, also called recall, measures the CAD’s capability to detect positive instances, while specificity measures the correctly detected proportion of true negatives. Higher specificity means that the system correctly identifies a higher value of the *TN*. Balancing sensitivity and specificity is important in the chosen classifier model, as we cannot optimize both simultaneously. Sensitivity is more affected by imbalanced datasets than specificity since it is based on the occurrence of the positive class. In contrast, specificity is based on the occurrence of the negative class.

The *F*1 score shows the harmonic mean of the precision and recall of a system. Similar to accuracy and other metrics, we must be careful while interpreting the *F*1 score because it may be high due to imbalanced precision and recall. Applications focusing on detecting all true positives at the expense of producing more false positives can use the F2 measure. In breast cancer detection and diagnosis applications, increasing false positives is not preferred since it will lead to detrimental medical procedures and treatments.

The ROC curve plots the recall versus the false-positive rate with a classification threshold value. A false-positive rate is calculated as

(6) $$FPR=1-Specificity=\frac{FP}{TN+FP}$$

A ROC curve visualizes the performance of the CAD system. The AUC value measures the area under the ROC curve, which is between 0 and 1, as 1 represents a perfect classifier model.

## 9. Discussions and Conclusions

Research in computer-aided detection and diagnosis systems using histopathology images has been trending over the last two decades. Figure 4 shows the percentage of detection/diagnosis methodologies used over the previous twelve years. The most commonly used methods are transfer learning, CNN/DCNN, and SVM. The trend of designing and implementing deep learning in all aspects of life created a shift away from knowledge-based systems. Deep learning methods are replacing knowledge-based approaches for a couple of reasons. Advancements in computing technologies allow researchers to train networks in acceptable time frames. An increase in public-domain databases makes it possible to employ supervised algorithms. Table 8 summarizes the reviewed works for histopathology images from 2010 to 2023 regarding preprocessing, segmentation, feature extraction, and classification methods.

This review summarized the CAD systems using breast histopathology images regarding datasets, preprocessing, segmentation, feature engineering, classification methods, and performance metrics between 2010 and 2022. The preprocessing stage mainly consisted of data augmentation to increase the size of the dataset to prevent overfitting during network training. Image transformations included rotations, reflections, scaling, and shearing. Color modifications were also made in the preprocessing due to variations in staining and acquisition methods. Segmentation is a significant stage for analyzing the region of interest (ROI), extracting distinct features, and characterizing and labeling the ROIs. Deep learning became popular in nucleus segmentation and detection. The popular segmentation methods were adversarial learning, K-mean clustering, deep convolutional networks, wavelet decomposition, and fuzzy C-mean algorithms. Feature engineering is an essential part of a CAD system, either hand-crafted by a knowledge-based system or automatically extracted by a deep network. Hand-crafted features were mainly based on morphology, color, and texture information. Only about 5% of the classifiers were unsupervised methods, including fuzzy logic. The remaining procedures were supervised methods, as transfer learning, CNNs, and SVMs were the popular choices. ResNet-18, ResNet-50, Inception V3, VGG-16, VGG-19, and AlexNet were used to improve the performance of the classification. Binary classification was studied more than multiclass classification.

Collecting medical information is challenging because of health information privacy and ethical reasons, and it requires immense time and effort. Therefore, it is difficult to establish balanced datasets. Current breast cancer histopathology image datasets vary in size, resolution, and image quality. Consequently, most studies employ augmentation methods to balance the datasets. Random zooming, cropping, and horizontal and vertical flips were performed to increase the database size or to balance the unbalanced datasets. Because the artificially generated images depend on the dataset’s existing images, it may lead to overfitting in deep learning models. One way to prevent overfitting is to only use the artificially generated images in the training stage. Another way to prevent overfitting is not to use augmentation methods to increase image datasets but to use transfer learning models. A recent study by Rana and Bhushan reported the results of a transfer learning model without using any augmentation methods [66]. They used seven transfer learning models, including LENET, VGG16, DarkNet53, DarkNet19, ResNet50, Inception, and Xception, on the BreakHis dataset. The best performance was achieved by Xception at 83.07%. The same work proposed a parameter for unbalanced datasets and achieved an accuracy of 87.17% with the DarkNet53 model.

We observed that there had been a significant decrease in research developing hand-crafted feature extraction techniques requiring expert-domain knowledge and deformable segmentation methods. At the same time, the number of deep learning-based models increased significantly. The advances in artificial intelligence and machine learning techniques will continue to attract researchers to design deep learning-based CAD systems. Image transformers have become an attractive approach to computer vision in recent years. They can be the next tool implemented in histopathology images. Deep learning models do not require content knowledge, expert input, or feedback other than the datasets labeled by experts. After the training stage, deep learning automatically extracts features to characterize the ROIs; however, it is a black-box approach, and it is unclear how those features are calculated and what they represent. Thus, it is important to pay extra attention while developing and using deep learning approaches, especially in healthcare applications. The fusion of expert knowledge and deep learning can be a solution to improve the confidence and performance of CAD systems.

As deep learning models are rapidly replacing knowledge-based CAD models, there is an urgent need for large breast cancer histopathology image datasets.

## Figures and Tables

**Table 1:** Most used publicly available histopathology image datasets and their corresponding URL.

Dataset Name	URL
The Breast Cancer Histopathological Image Classification (BreakHis)	https://www.kaggle.com/ambarish/breakhis (accessed on 28 April 2023.)
The Kaggle Breast Cancer Histopathology Images	https://www.kaggle.com/paultimothymooney/breast-histopathology-images (accessed on 28 April 2023.)
The ICIAR 2018 Grand Challenge on Breast Cancer Histology images (BACH)	https://iciar2018-challenge.grand-challenge.org/Dataset/ (accessed on 28 April 2023.)
Tumor Proliferation Assessment Challenge 2016 (TUPAC16)	https://github.com/DeepPathology/TUPAC16_AlternativeLabels (accessed on 28 April 2023.)
MITOS-ATYPIA-14 challenge	https://mitos-atypia-14.grand-challenge.org/Dataset/ (accessed on 28 April 2023.)
International Conference on Pattern Recognition (ICPR 2012) dataset	http://ludo17.free.fr/mitos_2012/download.html (accessed on 28 April 2023.)

**Table 2:** Image distribution by magnification factor and class for BreakHis dataset.

Magnification	Benign	Malignant	Total
×40	625	1370	1995
×100	644	1437	2081
×200	623	1390	2013
×400	588	1232	1820
Total images	2480	5429	7909

**Table 3:** Image distribution by magnification factor and class for the Kaggle dataset.

Magnification	Benign	Malignant	Total
×40	198,738	78,786	277,524

**Table 4:** Image distribution by class for ICIAR 2018 dataset.

Magnification	Normal	Benign	In Situ Carcinoma	Invasive Carcinoma	Total
×200	100	100	100	100	400

**Table 5:** Image distribution by class for the TUPAC16 dataset.

	Score 1	Score 2	Score 3	PAM50 Score (Mean ± STD)
Training	236 (47%)	117(23%)	147(30%)	−0.166 ± 0.446
Testing	147 (46%)	77(24%)	97(30%)	−0.192 ± 0.400

**Table 6:** Image distribution by class for the MITOS-ATYPIA-14 dataset.

Magnification	Number of Frames	Information
×20	284	Nuclear atypia score as a number 1, 2, or 3
×40	1136	Atypia scoring regarding the size of nuclei, size of nucleoli, the density of chromatin, thickness of the nuclear membrane, regularity of the nuclear contour, and anisonucleosis.

**Table 7:** Mitotic cell count distribution over different scanners used for the ICPR 2012 dataset.

Data Sets	Both Scanners	Multispectral Microscope
Training: 35 HPF	226	224
Evaluation: 15 HPF	100	98
Total	326	322

**Table 8:** Summary of the reviewed works between 2010 and 2023: preprocessing, segmentation, feature extraction, and classification methods. The table is arranged in chronological order.

Work Year	Dataset	Preprocessing	Segmentation	Features	Classifier	Performance
2023 [77]	BreakHis	-	-	Seven transfer learning models, VGG16, Darknet19, DarkNet53, LENET, ResNet50, Inception, and Xception	-	2-class: VGG16: 67.51% Darknet19: 80.57% DarkNet53: 70.59% LENET: 75.99% ResNet50: 81.85% Inception: 80.5% Xception: 83.09%
2023 [78]	BreakHis	-	-	Convolutional Neural Network, (2) a transfer learning architecture VGG16	Neural Network (64 units), Random Forest, Multilayer Perceptron, Decision Tree, Support Vector Machines, K-Nearest Neighbors, and Narrow Neural Network (10 units)	Magnification: 400× CNN achieved up to 85% for the Neural Network and Random Forest, the VGG16 method achieved up to 86% for the Neural Network
2022 [68]	Two public datasets and a new dataset: Bca-lym, Post-NAT-BRCA, TCGA-lym	-	Dense dual-task network (DDTNet)	Spatial and context cues, the multi-scale features with lymphocyte location information	All networks using Pytorch 1.1.0 and a NVIDIA GeForce RTX 2080 Ti GPU	Segmentation performance: Bca-lym dataset: Dice: 85.6% Post-NAT-BRCA dataset: Dice: 83.6% TCGA-lym dataset: Dice: 77.8%
2022 [40]	BreakHis BreCaHAD	Contrast-limited adaptive histogram equalization; Data augmentation	-	Ghost features	Stochastic Dilated Residual Ghost (SDRG) Model including ghost unit, stochastic downsampling, stochastic up-sampling units, and other convolution layers	BreakHis (x40) Original (93.13 ± 4.36) Augmented (98.41 ± 1.00) BreCaHAD Original (95.23 ± 4.38) Augmented (98.60 ± 0.99)
2022 [41]	BreakHis	Stain color normalization by Vahadane method; Random Zoom Augmentation with value 2, Random Rotation Augmentation with a value of 90° and Horizontal and Vertical Flip Augmentation	-	-	Three pre-trained deep convolutional neural networks work in parallel (xception, NASNet, and eptoin_resnet_V2)	The range of threshold values: 50–97% The range of accuracy depending on the threshold value: 96–98%
2022 [60]	Private dataset	Color augmentation, HE-stained and IHC-stained	Segmentation networks: Deeplab_v2, Linknet, Pspnet	-	Domain adaptation framework: Adversarial learning, Target domain data selection, Model refinement, Atrous Spatial Pyramid Pooling	Dice on HE: 87.9% Dice on IHC: 84.6%
2022 [62]	Private dataset of a total of 200 images at 10× magnification	Histogram matching algorithm for color normalization	Spatial neighborhood intensity constraint (SNIC) and knowledge-based clustering framework	Spatial information	K-Mean clustering algorithm	91.2%
2022 [70]	MITOS 2012 AMIDA 2013 MITOS 2014 TUPAC 2016	-	-	Three features vector sets Extended Local Pattern features, GLCM features from grayscale, GLCM features from V channel of HSV image	SVM, Random Forest Naïve Bayes Majority voting	MITOS 2012 Majority voting: F score: 95.64% MITOS 2014 Majority voting: F score: 86.38% AMIDA 13 Majority voting: F score: 73.09% TUPAC 16 Majority voting: F score: 78.25%
2022 [71]	DS1, DS2, DS3	-	-	Step-by-step valid convolutions	Input-collaborative PSV ConvNet	DS2: 90.4–93%
2022 [73]	BreakHis	Histogram Equalization	Otsu’s thresholding method using Red Channel	Geometrical Features Directional Features Intensity-based features	Decision Tree: Fine tree Linear SVM Fine KNN Narrow Neural Network (NNN)	2 class: NNN: 96.9%
2021 [72]	BreakHis	-	-	DCNN	Alexnet, VGG-16 Transfer learning methods, DCNN	2-class: 40×: 94% 100×: 95.45% 200×: 98.36% 400×: 85.71%
2021 [42]	BreakHis	Global contrast normalization; Three-fold data augmentation on training data	-	ResNet-18	Transfer learning based on block-wise fine-tuning strategy	MI classification: Binary: 98.42% Eight-class: 92.03% MD classification: Binary: 98.84% Eight-class: 92.15%
2021 [54]	The HUP 239 images, CINJ 40 images and TCGS 195, CWRU 110 images	Reduced image size, RGB to grayscale conversion, smoothing by Gaussian Filter	-	Unsupervised pre-training and supervised fine-tuning phase	Patch-based deep learning method called Pa-DBN-BC, Deep Belief Network (DBN), Logistic regressions	Overall: 86%
2021 [74]	BreaKHis	-	-	Convolution and capsule features Integrated sematic and special features	Deep feature fusion and enhanced routing, FE-BkCapsNet	2-class: 40×: 92.71% 100×: 94.52% 200×: 94.03% 400×: 93.54%
2021 [79]	BreaKHis	Color normalization technique	-	Feature Extraction-Based CML Approaches, Zernike moments, Haralick, and color histogram features	Conventional machine learning (CML) and deep learning (DL)-based methods	2-class: DL: 94.05–98.13% CML: 85.65–89.32% 8-class: DL: 76.77–88.95% CML: 63.55–69.69%
2020 [67]	Two small datasets: 50 images of 11 patients; 30 H&E marked 40× magnified images	Median filter, Bottom + Top Hat filter	Identifying thresholds based on the energy curve, finding the best threshold using the entropy	Area, major axis length, minor axis length	-	Dataset 1: 93.1% Dataset 2: 93.5%
2020 [76]	BACH dataset	Data augmentation by color normalization, vertical and horizontal mirroring, random rotations, addition of random noise and random change in intensity of the images	-	CNN-based feature extraction network	Region Guided Soft Attention	90.25%
2020 [80]	BACH 2018	-	-	Indexes based on phylogenetic diversity.	SVM, Random Forest MLP XGBoost	4-class: 95%
2020 [55]	Private dataset of 8009 histopathology images from over 683 patients with different magnification levels	Gaussian filtering technique for noise removal, data augmentation by rotation	Histo-sigmoid-based fuzzy clustering	-	Deep Neural Network with Support Value (DNNS)	97.21%
2020 [44]	Private dataset	Data augmentation	-	Multi-level and multiscale deep features	Ensemble of fine-tuned VGG16 and fined tuned VGG19	Up to 95.29%
2020 [52]	BreakHis	Data augmentation, random horizontal flip, color jitter, random rotation, and crop	-	Feature maps	Deep transfer learning-based models: DensNet and ResNet, ResNet101, VGG19, AlexNet, and SqueezeNet	2-class: BreakHis (40×): 100% BreakHis (100×): 100% BreakHis (200×): 98.08% BreakHis (400×): 98.99% Multi-class: BreakHis (40×): 97.96% BreakHis (100×): 97.14% BreakHis (200×): 95.19% BreakHis (400×): 94.95%
2020 [61]	Private dataset consists of 428 images from 240 breast biopsies	-	Ductal Instance-Oriented Pipeline (DIOP) segmentation model: a duct-level instance segmentation model, tissue-level semantic segmentation model, three levels of features	Histogram features Co-occurrence features Structural features	Random forest model, 3-degree polynomial SVM SVM-RBF Multilayer perception with four hidden layers	2-class: Invasive vs. non invasive: 95% Atypia and DCIS vs Benign: 79% DCIS vs. Atypia: 90% Multi-class: 70%
2020 [63]	ICPR 2012 MITOSIS Dataset, 2014 ICPR dataset, and the AMIDA13 dataset	-	Segmentation branch trained with weak and strong labels	Convolution features	Pre-trained and fine-tuned Partially supervised framework based on two parallel, deep fully convolutional networks	2012 ICPR MITOSIS dataset F-scores: 0.788 2014 ICPR dataset: F-scores: 0.575 AMIDA13 dataset: F-scores: 0.698
2020 [64]	Dataset of 640 H&E-stained breast histopathology images	Data augmentation by random zooming, cropping, horizontal and vertical flips	A tile-wise segmentation strategy, (a) direct tile-wise merging; (b) tile-wise merging based on a Conditional Random Field (CRF)	-	DCNN-based architecture	Xception 65: 95.62% Mobilenet v2: 92.9% Resnet v1: 91.16%
2019 [45]	-Bioimaging-2015 -BreakHis	Stain color normalization; Logarithmic transformation; Data Augmentation	-	Ensemble of DCNNs	Gradient boosting trees classifier	Bioimaging-2015 (4-class): 96.4% Bioimaginf-2015 (2-class): 99.5% BreakHis (40×): 95.1% BreakHis (100×): 96.3% BreakHis (200×): 96.9% BreakHis (400×): 93.8%
2019 [46]	ICIAR 2018 BreakHis	Stain color normalization; Image decomposition via Haar wavelet; Data Augmentation	-	Deep features from Haar wavelet decomposed images by a CNN model; Incorporation of multiscale discriminant features	Three fully connected two Dropout and SoftMax layers	ICIAR 2018 (2 and 4-class): 98.2% BreakHis (Multi-class): 96.85%
2019 [50]		Data Augmentation	-	Feature vectors	CNN with IDC patch-based classification	85.41%
2019 [57]	BreakHis	Contrast enhancement by histogram Equalization, color constancy	-	CNN features	5 Convolutional layers Fully connected and SoftMax layer	Hist. Equalization with the proposed method: AUC: 87.6% Color constancy with the proposed method: AUC: 93.5%
2019 [58]	Bioimaging Challenge 2015	Singular value decomposition (SVD), Logarithmic transformation	-	CNN based on the SE-ResNet module GoogleNet, Xception, Inception-ResNet, 3-Norm pooling method	KNN SVM	SVM-GoogleLeNet 2-class: 91.67% 4-class: 83.33%
2019 [69]	TUPAC 16 MITOS12 + MITOS14	Stain normalization Annotation Cropping	Transfer Learning-based Mitosis Segmentation (TL-Mit-Seg)	-	Hybrid-CNN based mitosis detection module (HCNN-Mit-Det); HCNN-Mit-Det-essemble; Transfer learning HCNN-Mit-Det	TUPAC 16: F-measure: 66.7% MITOS12 + MITOS14 F-measure: 65.1%
2018 [48]	ICIAR 2018	Data Augmentation: 50 random color augmentations; different image scales	-	ResNet-50, InceptionV3 and VGG-16 networks from Keras distribution	Gradient boosted trees classifier	2-class: 93.8% 4-class: 87.2%
2017 [51]	BreakHis	Data Augmentation randomly distorted images, rotated and mirrored images	-	Transfer learning Google Inception v3	Deep convolutional neural network(CNN, ConvNet) model	83% for benign class 89% for malignant class
2015 [65]	Private dataset of 100 malignant and nonmalignant breast histology images	-	Spatial-color-texture-based graph partitioning method	Intensity-texture features Color texture features	-	
2015 [66]	68 BCH images containing more than 3600 cells.	Top-bottom hat transform	Wavelet decomposition and multiscale region growing	4 shape-based features and 138 textural features based on color spaces, wrapper feature selection algorithm based on chain-like agent genetic algorithm (CAGA)	SVM	Normal vs. malignant: 96.19 ± 0.31%

## Data Availability

The text and references include links to publicly archived datasets.
